# Endoscopic assessment of minor papilla morphology: Predictors of successful cannulation and procedural pancreatitis risk in minor papilla endotherapy

**DOI:** 10.1002/jhbp.12068

**Published:** 2024-09-09

**Authors:** Yasuhiro Kuraishi, Akira Nakamura, Shohei Kondo, Takumi Yanagisawa, Ichitaro Horiuchi, Masafumi Minamisawa, Nobukazu Sasaki, Yugo Iwaya, Tadanobu Nagaya, Takeji Umemura

**Affiliations:** ^1^ Department of Gastroenterology Shinshu University Hospital Nagano Japan

**Keywords:** cannulation success, endoscopic appearance, endoscopic retrograde cholangiopancreatography, minor papilla, post‐ERCP pancreatitis

## Abstract

**Background:**

We evaluated for predictors of successful cannulation and post‐endoscopic retrograde cholangiopancreatography (ERCP) pancreatitis (PEP) in minor papilla endotherapy (MPE), emphasizing endoscopic minor papilla morphology.

**Methods:**

We retrospectively analyzed 232 MPEs in 65 patients, assessing minor papilla morphology based on three features: bulge as “prominent” or “subtle,” mucosal appearance as “papilla‐like” resembling the main papilla or “SMT‐like” akin to a gastrointestinal submucosal tumor, and orifice visibility as “clear” or “unclear.” Cannulation success was evaluated in 65 enrolled patients, with PEP risk assessed in all 232 MPEs.

**Results:**

Minor papilla morphology was categorized as prominent/subtle bulge in 42/23 patients, papilla‐like/SMT‐like mucosal appearance in 42/23, and clear/unclear orifice visibility in 24/41. Cannulation succeeded in 54/65 patients (83%). A papilla‐like appearance and clear orifice visibility was significantly associated with cannulation success. PEP incidence was 5.2% and predominantly mild. A papilla‐like appearance significantly decreased PEP incidence, while precutting technique and orifice dilation significantly increased PEP risk.

**Conclusion:**

Evaluating minor papilla morphology may help predict cannulation success and PEP risk in MPE. A papilla‐like mucosal appearance prognosticates cannulation success and reduced PEP risk, with clear orifice visibility serving as a success predictor. These findings provide practical guidance for preprocedural planning by emphasizing the importance of minor papilla morphology evaluation.

## Graphical abstract

Kuraishi and colleagues demonstrated the usefulness of endoscopic assessment of minor papilla morphology in predicting cannulation success and the likelihood of complications following minor papilla endotherapy, providing critical guidance for planning. In particular, a papilla‐like mucosal appearance and clear orifice visibility significantly predicted successful cannulation, reducing the risk of post‐ERCP pancreatitis.

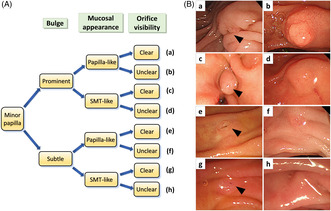

## 1 INTRODUCTION

Endoscopic retrograde cholangiopancreatography (ERCP) plays a prominent role in diagnosing and treating pancreatic disorders, mainly by accessing the main pancreatic duct (MPD) via the major papilla. However, when this approach is untenable due to conditions such as pancreatic divisum, Wirsung's duct distortion, and obstruction, an alternative route through the minor papilla is needed. Minor papilla endotherapy (MPE) has demonstrated efficacy, particularly for pancreatic divisum, with a meta‐analysis revealing significant clinical improvement in both recurrent (76%) and chronic (52%) pancreatitis post‐MPE.^1^ Despite its utility in cases unmanageable through the major papilla,^2^ however, the application of MPE is largely confined to specific conditions, which limits its clinical utilization and reported findings.

The pursuit of successful and safe MPE faces several challenges, notably in accessing the MPD and managing the risk of procedural complications. Cannulation of the minor papilla is particularly challenging, even for skilled endoscopists, due to its diminutive opening.^3^, ^4^ Techniques such as the rendezvous^5^, ^6^ and precutting^7^, ^8^ methods have been developed to mitigate these access difficulties. Special attention is also warranted for post‐ERCP pancreatitis (PEP), the most prevalent and serious procedure‐related complication with a prevalence of 5%–35%.^3^, ^9^, ^10^, ^11^, ^12^, ^13^, ^14^, ^15^, ^16^, ^17^ MPE procedures carry a higher risk of PEP over standard ERCP techniques through the major papilla.^9^ Repeated cannulation attempts also increase the risk of PEP,^18^, ^19^ with a clear link between cannulation difficulty and PEP incidence in standard ERCP procedures.^20^, ^21^ This association highlights the need for careful and precise cannulation planning to minimize the complications associated with MPE.

The minor papilla displays wide variability in its endoscopic appearance among individuals. Assessing its morphology, such as minor papilla bulge and orifice visibility, reportedly provides predictive information on the existence of pancreatic duct abnormalities.^22^ The relationship between the endoscopic morphology of the major papilla and cannulation difficulty is well‐documented,^23^, ^24^, ^25^ suggesting that similar assessments of minor papilla morphology can offer valuable insights into the complexities involved in its cannulation and assist in devising effective strategies for accessing the MPD. However, no reports have addressed the relationship between endoscopic minor papilla appearance and cannulation success or procedure‐related complications in MPE. The present study explored the factors predicting successful cannulation rate and the PEP prevalence in MPE, with a particular focus on the endoscopic characteristics of the minor papilla.

## 2 METHODS

### 2.1 Patients

Between January 2008 and October 2023, a total of 1794 ERCP procedures accessing the MPD were performed on 739 patients at Shinshu University Hospital for the diagnostic and therapeutic management of pancreatic pathologies. This retrospective study focused on 237 ERCP cases involving MPE in 70 patients identified through a comprehensive review of electronic medical records. Patients undergoing MPD access via the rendezvous cannulation technique were excluded to target the impact of minor papilla morphology on cannulation success. The investigation adhered to the ethical guidelines of the Declaration of Helsinki and the Institutional Review Board of Shinshu University Hospital (approval no. 6044). Written informed consent was obtained from all participants prior to the ERCP procedures.

### 2.2 Outcome measurement

The primary outcome of interest was factors predicting successful cannulation of the minor papilla with an emphasis on its endoscopic appearances. Cannulation success was assessed in patients with a naïve minor papilla who had not previously undergone MPE procedures. Cannulation failure was recorded when at least one attempt at cannulation was unsuccessful at any point during the procedure.

The secondary outcome of interest was risk factors for PEP in MPE procedures. ERCP‐related adverse events were defined according to consensus criteria.^26^ Following the procedure, patients were hospitalized for at least 24 h for monitoring procedural complications. Laboratory examinations were conducted on the next day, and PEP was identified as an elevation in serum amylase of at least threefold above the normal range accompanied by abdominal pain. Hyperamylasemia was characterized as a serum amylase elevation of at least three times the normal level, irrespective of the presence or absence of abdominal pain. The risk factors for PEP were analyzed for all MPE procedures irrespective of a naïve status of the minor papilla.

**FIGURE 1:**
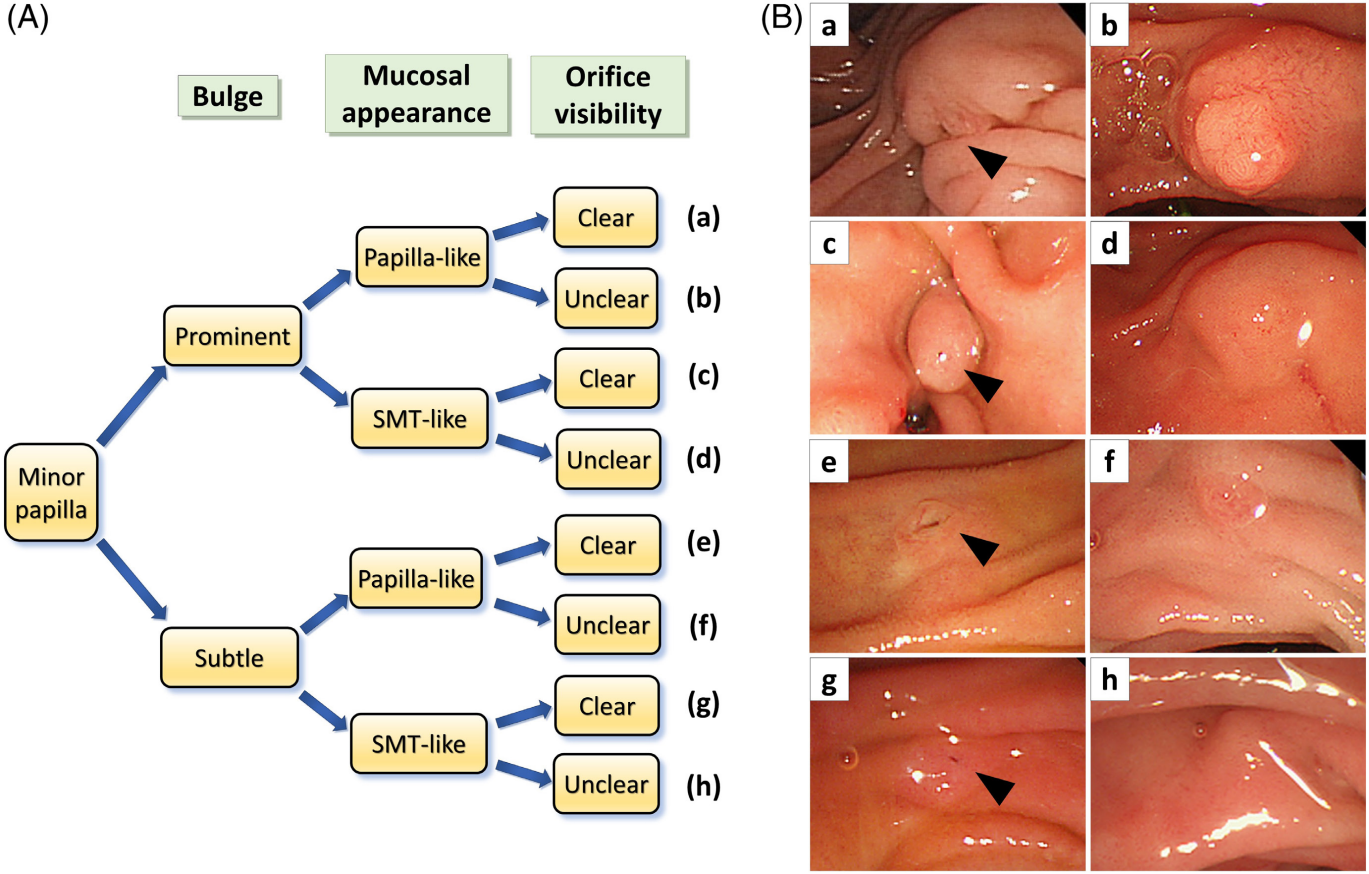
(A) Classification of minor papilla morphology. Minor papilla morphology was assessed precannulation focusing on three main features: Bulge as “prominent” or “subtle,” mucosal appearance as “papilla‐like” by resembling the main papilla or “SMT‐like” akin to a gastrointestinal submucosal tumor, and orifice visibility as “clear” or “unclear.” (B) Representative endoscopic images of the minor papilla. Our classification divided the minor papilla into eight categories, represented as endoscopic images (a–h). Arrowheads indicate the orifice.

We classified minor papilla morphology using precannulation endoscopic images by focusing on three main features: minor papilla bulge, mucosal appearance surrounding the orifice, and orifice visibility (Figure 1). The morphology of the minor papilla was assessed using the endoscopic white light imaging. The extent of mound bulging was deemed “prominent” when its height exceeded the surrounding duodenal folds and “subtle” when less pronounced. Mucosal appearance was categorized as “papilla‐like” when resembling the main papilla with a distinct papillary structure or concentric grooves or “SMT‐like” when akin to a submucosal tumor in the gastrointestinal tract, featuring a flat or smooth surface with blurred boundaries with the surrounding mucosa. Orifice visibility was classified as “clear” if a gaping orifice or visibly dripping pancreatic juice was observed and “unclear” if no visible orifice was present. The evaluation of minor papilla morphology was conducted by reviewing still images captured during the endoscopic procedures. Two experienced endoscopists independently assessed the images to classify morphology based on the above criteria. If their evaluations differed, a third endoscopist examined the endoscopic image, and the morphology was determined through consensus among all three reviewers. The endoscopists were blinded to the results of cannulation success and the incidence of PEP. Other parameters potentially influencing cannulation success and complications also were evaluated, including gender, age, underlying pancreatic conditions, diameter and tortuosity of Santorini's duct, scope position when cannulating the minor papilla (long route or short route position), in addition to such ERCP procedure characteristics as the occurrence of major papilla cannulation, precutting, sphincterotomy, orifice dilation, stent placement, and stone removal. Santorini's duct was assessed by ERP or magnetic resonance cholangiopancreatography.

### 2.3 Procedures

Patients underwent MPE procedures in a prone position using a side‐viewing duodenoscope (JF‐240, JF‐260V, or TJF‐Q290V; Olympus Optical Co., Ltd., Tokyo, Japan). Sedation was maintained intravenously with midazolam and pethidine in addition to hyoscine butylbromide to relax the duodenum. Prophylactic nonsteroidal anti‐inflammatory drug suppositories were administered to prevent PEP.^27^ Cannulation techniques varied in terms of cannulas, sphincterotomes, and guidewires, with a preference for the guidewire‐assisted method using a tapered cannula preloaded with a 0.025‐inch guidewire. An angle‐tip guidewire was used for cannulation due to its superior maneuverability within the pancreatic duct, facilitating navigation through ductal structures and tortuous anatomy. We adjusted the scope position from the short to the long route in order to optimize alignment between the minor papilla and the scope based on individual anatomical variations and procedural needs. In cases where locating the minor papilla orifice was difficult, an indigo carmine spray was applied to facilitate its detection. For challenging cannulations, salvage techniques like the rendezvous or precutting methods were employed. Direct cannulation of the minor papilla was initiated in cases preidentified with pancreatic divisum, while ERCP attempts initially targeted the major papilla in the absence of divisum. Conditions related to Wirsung's duct distortion or obstruction that hindered access to the upstream MPD necessitated a strategic pivot to cannulate the minor papilla. Following successful access, such interventions as sphincterotomy and balloon dilation were conducted based on each case's requirements. The selection of instruments and procedural strategies was tailored by the endoscopist to each situation.

### 2.4 Statistical analysis

For data analysis, categorical variables are presented as counts and percentages, while continuous variables are summarized as medians with ranges. Pearson's chi‐squared test was used to assess differences between subgroups, providing the odds ratio (OR) and a 95% confidence interval (CI). Statistical significance was defined as a *p*‐value of less than .05. Interobserver agreement between two endoscopists in the assessment of minor papilla morphology was measured using Cohen's kappa coefficient. The results were interpreted based on the following classification: slight (0.01–0.20), fair (0.21–0.40), moderate (0.41–0.60), substantial (0.61–0.80), and almost perfect (0.81–1.00). All statistical analyses were performed using JMP Statistics software version 13 (JMP, Tokyo, Japan).

## 3 RESULTS

### 3.1 Patients and procedures

During the study period, 237 MPE procedures were performed on 70 patients. Excluding five cases employing the rendezvous cannulation technique, 232 procedures on 65 patients (52 male and 13 female) were included for analysis. Clinical characteristics included a median age of 64 years (range: 22–82 years) and a median of two procedures per patient (Table 1). The primary indications for accessing the minor papilla were pancreatic divisum (*n* = 20), followed next by obstruction (*n* = 17) and distortion of Wirsung's duct (*n* = 15). Chronic pancreatitis was the most common underlying condition (*n* = 40), along with pancreatic divisum (*n* = 20), autoimmune pancreatitis (*n* = 10), pancreatic pseudocyst (*n* = 9), and pancreatic tumor (*n* = 7).

**TABLE 1:** Patient characteristics.

	*N* = 65
Gender (male/female), *n*	52/13
Age (years), median [range]	64 [22–82]
Number of procedures per patient, median [range]	2 [1–23]
Pancreatic disease, *n* (%)	
Chronic pancreatitis	40 (62)
Pancreatic divisum	20 (31)
Autoimmune pancreatitis	10 (15)
Pancreatic pseudocyst	9 (14)
Pancreatic tumor	7 (11)
Reason for access through minor papilla, *n* (%)	
Pancreatic divisum	20 (31)
Obstruction or stenosis of Wirsung's duct	17 (26)
Distortion of Wirsung's duct	15 (23)
Failed cannulation through major papilla	3 (5)
Other	10 (15)
Endoscopic minor papilla appearance, *n*	
Bulging mound: prominent/subtle	42/23
Mucosal appearance: papilla‐like/SMT‐like	42/23
Visibility of orifice: clear/unclear	24/41
Diameter of Santorini's duct (mm), median [range]	2 [0.3–6.2]
Tortuous Santorini's duct, *n* (%)	22 (34)
Scope position (long route/short route), *n* (%)	46/19
Successful cannulation through minor papilla, *n* (%)	54 (83)

Abbreviation: SMT, submucosal tumor.

Endoscopic evaluation of the minor papilla showed “prominent” bulges in 42 patients and “subtle” bulges in 23 patients. Mucosal appearance was classified as “papilla‐like” in 42 patients and “SMT‐like” in 23 patients, with orifice visibility judged as “clear” in 24 patients and “unclear” in 41 patients. The interobserver agreements in the classification of minor papilla morphology between two endoscopists were almost perfect for assessing the minor papilla bulge (*κ* = 0.87), and substantial for evaluating mucosal appearance (*κ* = 0.63) and orifice visibility (*κ* = 0.80). Santorini's duct measurements revealed a median diameter of 2 mm (range: 0.3–6.2 mm), with 22 patients exhibiting tortuous anatomy. When cannulating the minor papilla, the long route scope position was adopted in 46 patients and the short route scope position was employed in 19 patients. Successful cannulation of the minor papilla was achieved in 54 of 65 patients (83%), which was during the first ERCP session in 52 patients and in the second session in two patients. In the 11 remaining patients, the cannulation procedure was deemed unsuccessful after a single attempt in nine patients and following multiple attempts in two patients. To identify the minor papilla orifice, an indigo carmine spray was used in three patients.

Table 2 summarizes the procedural details of the 232 MPE procedures. Before minor papilla cannulation, 114 ERCPs (49%) attempted access through the major papilla. Precutting cannulation techniques were employed in 16 ERCPs (7%). Among the MPE procedures, specific interventions included minor papilla sphincterotomy in 33 cases (14%), orifice dilation in 21 cases (9%), plastic stent placement in 113 cases (49%), nasopancreatic drainage tube placement in 18 cases (8%), and stone extraction in 56 cases (24%).

**TABLE 2:** Procedure characteristics.

	*N* = 232
Naïve minor papilla, *n* (%)	87 (38)
Attempt of major papilla cannulation, *n* (%)	114 (49)
Minor papilla precutting technique, *n* (%)	16 (7)
Minor papilla sphincterotomy, *n* (%)	33 (14)
Minor papilla orifice dilation, *n* (%)	21 (9)
Pancreatic stent placement, *n* (%)	113 (49)
Nasopancreatic drainage tube placement, *n* (%)	18 (8)
Stone extraction through minor papilla, *n* (%)	56 (24)
Biopsy through minor papilla, *n* (%)	2 (0.8)
Brushing cytology through minor papilla, *n* (%)	10 (4)

### 3.2 Factors predicting cannulation success

We next analyzed for factors predicting cannulation success in the 65 patients with a naïve minor papilla, with a special focus on the three above‐described endoscopic features (Table 3). A papilla‐like mucosal appearance was significantly associated with cannulation success as compared with a SMT‐like appearance (93% vs. 65%; OR 6.93, 95% CI: 1.62–29.7, *p* = .005). Clear orifice visibility also significantly associated with cannulation success versus unclear orifice visibility (96% vs. 73%; OR 8.43, 95% CI: 1.01–70.1, *p* = .048). No relationship was detected between the extent of minor papilla bulge and cannulation outcome. A Santorini's duct diameter ≥2 mm indicated a tendency for successful cannulation, albeit not significantly. Other patient factors, such as gender, age, underlying pancreatic disease, Santorini's duct tortuosity, and scope position, were not significantly related to success rate.

**TABLE 3:** Predictive factors of cannulation success.

Variable	*N*	Cannulation success *n* (*n*/*N*, %)	OR (95% CI)	*p*‐value
Patient characteristics				
Gender				
Male	52	45 (87)	2.86 (0.69–11.8)	.137
Female	13	9 (69)	Reference	
Age				
≥65 years	29	23 (79)	0.62 (0.17–2.28)	.467
<65 years	36	31 (86)	Reference	
Chronic pancreatitis				
Yes	40	35 (88)	2.21 (0.60–8.21)	.229
No	25	19 (76)	Reference	
Pancreatic divisum				
Yes	20	17 (85)	1.23 (0.29–5.20)	.783
No	45	37 (82)	Reference	
Autoimmune pancreatitis				
Yes	10	7 (70)	0.40 (0.08–1.86)	.231
No	55	47 (86)	Reference	
Santorini's duct diameter				
≥2 mm	35	32 (91)	3.88 (0.92–16.3)	.053
<2 mm	30	22 (73)	Reference	
Tortuous Santorini's duct				
Yes	22	16 (73)	0.35 (0.09–1.32)	.111
No	43	38 (88)	Reference	
Scope position				
Long route	46	39 (85)	1.48 (0.38–5.82)	.570
Short route	19	15 (79)	Reference	
Endoscopic minor papilla appearance				
Bulging mound				
Prominent	42	37 (88)	2.61 (0.70–9.76)	.145
Subtle	23	17 (74)	Reference	
Mucosal appearance				
Papilla‐like	42	39 (93)	6.93 (1.62–29.7)	.005
SMT‐like	23	15 (65)	Reference	
Visibility of orifice				
Clear	24	23 (96)	8.43 (1.01–70.1)	.048
Unclear	41	30 (73)	Reference	

Abbreviations: 95% CI, 95% confidence interval; OR, odds ratio; SMT, submucosal tumor.

### 3.3 Complications and risk factors of PEP

Of the 232 MPE procedures, 12 cases (5.2%) of PEP were recorded along with 27 cases of hyperamylasemia (12%) (Table 4). The majority of PEP cases were mild (11 episodes), with one moderate case and no instances of severe PEP. Mild bleeding following minor papilla sphincterotomy was recorded in one case, which was successfully managed by endoscopic hemostasis without the need for transfusion. There were no procedure‐related perforations and fatalities.

**TABLE 4:** ERCP‐related complications.

	*N* = 232
Pancreatitis, *n* (%)	12 (5.2)
Mild	11
Moderate	1
Severe	0
Hyperamylasemia, *n* (%)	27 (12)
Bleeding, *n* (%)	1 (0.4)
Perforation, *n* (%)	0 (0)
Death, *n* (%)	0 (0)

Abbreviation: ERCP, endoscopic retrograde cholangiopancreatography.

Factors affecting PEP development were examined in all 232 MPE procedures (Table 5). A papilla‐like mucosal appearance had a significantly lower incidence of PEP (3.5%) as compared with a SMT‐like appearance (10%) (*p* = .049). The other minor papilla morphologies showed no significant impact on PEP risk. The likelihood of PEP was significantly higher in procedures involving a naïve minor papilla (9.2%) than in those without (2.8%) (*p* = .032). Patients with chronic pancreatitis had a significantly lower incidence of PEP (3.7%) than did those without (11.6%) (*p* = .034). Moreover, procedures that utilized the precutting technique for cannulation (25.0% PEP incidence) and minor papilla orifice dilation (14.3% PEP incidence) were associated with a significantly higher incidence of PEP versus procedures not employing those methods (3.7% and 4.3% PEP incidence, respectively) (*p* < .001 and *p* = .048, respectively). The use of minor papilla sphincterotomy (12.1%) showed a trend toward increasing PEP incidence as compared with procedures that did not employ this technique (4.0%) (*p* = .052). No significant differences were detected for PEP occurrence in relation to gender, age, Santorini's duct diameter, or the involvement of other specific MPE procedures.

**TABLE 5:** Risk factors for PEP development.

Variable	*N*	PEP *n* (*n*/*N*, %)	OR (95% CI)	*p*‐value
Patient characteristics				
Gender				
Male	210	9 (4.3)	0.28 (0.07–1.14)	.060
Female	22	3 (13.6)	Reference	
Age				
≥65 years	109	4 (3.7)	0.54 (0.16–1.87)	.331
<65 years	123	8 (6.5)	Reference	
Chronic pancreatitis				
Yes	189	7 (3.7)	0.29 (0.09–0.97)	.034
No	43	5 (11.6)	Reference	
Pancreatic divisum				
Yes	78	6 (7.7)	2.06 (0.64–6.60)	.217
No	154	6 (3.9)	Reference	
Naïve minor papilla				
Yes	89	8 (9.2)	5.72 (1.66–19.7)	.032
No	145	4 (2.8)	Reference	
Santorini's duct diameter				
≥2 mm	81	4 (4.9)	0.93 (0.27–3.18)	.906
<2 mm	151	8 (5.3)	Reference	
Endoscopic minor papilla appearance				
Bulging mound				
Prominent	151	9 (6.0)	1.64 (0.43–6.27)	.459
Subtle	81	3 (3.7)	Reference	
Mucosal appearance				
Papilla‐like	174	6 (3.5)	0.32 (0.10–0.98)	.049
SMT‐like	60	6 (10)	Reference	
Visibility of orifice				
Clear	58	4 (6.2)	1.30 (0.38–4.49)	.674
Unclear	176	8 (4.8)	Reference	
ERCP procedure characteristic				
Major papilla cannulation				
Attempted	114	8 (7.0)	2.15 (0.63–7.35)	.212
Not attempted	118	4 (3.4)	Reference	
Precutting technique				
Yes	16	4 (25.0)	8.67 (2.28–32.9)	<.001
No	216	8 (3.7)	Reference	
Minor papilla sphincterotomy				
Yes	33	4 (12.1)	3.29 (0.93–11.6)	.052
No	199	8 (4.0)	Reference	
Minor papilla orifice dilation				
Yes	21	3 (14.3)	3.74 (0.93–15.1)	.048
No	211	9 (4.3)	Reference	
Pancreatic stent placement				
Yes	113	5 (4.4)	0.74 (0.23–2.41)	.616
No	119	7 (5.9)	Reference	
Stone extraction				
Yes	56	2 (3.6)	0.61 (0.13–2.89)	.535
No	176	10 (5.7)	Reference	

Abbreviations: 95% CI, 95% confidence interval; ERCP, endoscopic retrograde cholangiopancreatography; OR, odds ratio; PEP, post‐ERCP pancreatitis; SMT, submucosal tumor.

## 4 DISCUSSION

With a particular focus on the endoscopic evaluation of minor papilla appearance, the current investigation identified several factors significantly impacting cannulation success and PEP risk in MPE. These findings offer novel clinical insights that may improve the safety and efficacy of MPE procedures.

Significant associations were detected between the endoscopic morphology of the minor papilla and cannulation success. Specifically, we observed that a papilla‐like mucosal appearance and clear visibility of the orifice were significant indicators of successful cannulation. These results suggest that visual cues from endoscopic images can serve as valuable guidance for endoscopists in selecting an appropriate strategy for accessing the MPD. A minor papilla presenting with a SMT‐like mucosal appearance likely exhibits pliable and nonelastic properties, rendering it prone to deformation during cannulation attempts with an ERCP cannula. This may complicate the precise alignment required between the Santorini's duct's pathway and the orientation of the cannula or guidewire to hamper the cannulation process. The orifice of the minor papilla is typically smaller than that of the major papilla, leading to difficulty in locating its opening as a main reason for cannulation failure during MPE. Consequently, clear orifice visibility, although less common, is advantageous for successful cannulation.

Our study revealed an 83% cannulation success rate, which corroborated previous reports of 74% to 96%.^3^, ^9^, ^10^, ^11^, ^12^, ^13^, ^14^, ^15^, ^16^, ^17^ Prior investigations incorporated cases with non‐naïve papilla and predominantly included patients with pancreatic divisum, which implied reduced cannulation difficulty owing to factors like post‐procedural orifice enlargement or inherently well‐developed papilla. In contrast, our cohort contained naïve papilla and a variety of pancreatic conditions beyond pancreatic divisum, offering a more comprehensive insight into the real‐world challenges and outcomes associated with MPE procedures. While previous studies identified pancreatic calcification as a factor leading to cannulation failure,^13^ our analysis revealed no significant correlation between cannulation success and specific pancreatic disorders, possibly reflecting a variance in patient demographics and clinical contexts. The established correlation between the morphology of the major papilla and cannulation success, which underscores an association with endoscopic features and cannulation challenges,^23^, ^24^, ^25^ has not yet been explored for the minor papilla in the context of MPE. As the minor papilla cannulation presents unique challenges,^3^, ^4^ assessing its morphology represents an important step in determining MPE feasibility.

In addition to cannulation success, we addressed the issue of PEP risk, a significant complication in ERCP‐related procedures. Whereas the reported PEP incidence ranges broadly between 5% and 35%,^3^, ^9^, ^11^, ^12^, ^13^, ^14^, ^15^, ^16^, ^17^ our MPE procedures exhibited a relatively low rate of 5.2%, predominantly involving mild cases. The endoscopic morphology of the minor papilla may have impacted PEP risk, with a papilla‐like mucosal appearance significantly associated with decreased PEP incidence. This finding suggests that endoscopic assessment of minor papilla appearance not only aids in predicting cannulation success, but also helps prognosticate post‐procedural complications.

Our investigation also identified several procedural factors influencing PEP onset. Use of the precutting cannulation technique and minor papilla dilation were both associated with a higher incidence of PEP. Minor papilla sphincterotomy has also been reported as a PEP risk factor in MPE,^28^ which was corroborated by our findings, showing an increased trend of PEP associated with this procedure. The higher rates of PEP with sphincterotomy and orifice dilation may stem from their invasive approach to enlarge the minor papilla orifice. The established link between precutting and PEP risk in the context of cannulation difficulty^18^, ^19^, ^20^, ^21^ supports our findings. Moreover, we observed a higher incidence of PEP in cases displaying a SMT‐like mucosal appearance and in procedures involving a naïve minor papilla, both of which indicative of challenging cannulation conditions. In contrast, the presence of chronic pancreatitis acted as a protective factor against PEP, consistently with existing studies.^28^, ^29^ Taken together, our analysis underscores the importance of careful risk assessment in MPE procedures to optimize patient outcomes while mitigating potential complications.

ERCP‐related procedures such as MPE are the first line of endotherapy in patients with symptomatic MPD obstruction. Endoscopic ultrasound pancreatic duct drainage (EUS‐PDD) can be employed as a rescue technique for MPD access when the endoscopist is unable to achieve successful canulation in MPE.^30^, ^31^ In a study evaluating the endoscopic outcomes following an ERCP approach and EUS‐PDD in patients with benign MPD obstruction, the addition EUS‐PDD to ERCP improved the technical success rates of endoscopic interventions from 77% to 91%, with an overall clinical success rate of 97% in patients who underwent successful endotherapy.^30^ In the present investigation, we observed that patients with an SMT‐like mucosal appearance or an unclearly visible orifice had lower cannulation success rates. For these cases, insisting on minor papilla cannulation may not be the optimal strategy; instead of repeated ERCP attempts, early transition to EUS‐PDD should be considered as an effective and safe strategy in MPE to mitigate the risks associated with difficult cannulation and improve overall procedural outcomes.

Our study had several limitations. First, the classification of minor papilla morphology may have been subjective and lacked standardization due to the absence of a pre‐established classification system. To enhance interobserver agreement, however, we provided clear and concise definitions of each two states, thus improving objectivity and reproducibility. The interobserver agreements of minor papilla morphology classification were almost perfect for assessing the minor papilla bulge (*κ* = 0.87), and substantial for evaluating mucosal appearance (*κ* = 0.63) and orifice visibility (*κ* = 0.80). These results suggest that this classification is clinically acceptable for use as a decision‐making tool in MPE. Second, the retrospective nature of our data could have introduced selection bias. Third, this study contained a relatively small sample size of MPE procedures obtained from a single‐center and might lack generalizability. Due to the limited sample size of our cohort, the number of events, including unsuccessful cannulation and PEP occurrence, was insufficient to conduct a reliable multivariate analysis. Fourth, we evaluated the endoscopic morphology of the minor papilla using standard white light imaging only, without such image enhancement endoscopy (IEE) technologies as narrow band imaging or texture and color enhancement imaging. Although white light imaging is widely available and routinely used in the clinical setting, emerging IEE methods have the potential to improve assessment accuracy and should be considered in future studies. Moreover, ascertaining the proficiency of the original operator, whether an expert or a trainee, was not possible due to potential changes in personnel as the procedure went on. This issue was also compounded by the retrospective nature of our study. Despite the inherent difficulties in conducting a large‐scale randomized controlled trial in this specialized field, validation and broader application of our results will benefit from future prospective studies across multiple centers.

In conclusion, our study highlighted a role of minor papilla endoscopic assessment in predicting cannulation success and the likelihood of complications following MPE. Specifically, the presence of a papilla‐like mucosal appearance may promote cannulation success and decrease the risk of PEP, with a clear orifice also representing a predictor of successful cannulation. These endoscopic insights provide practical guidance for MPE planning toward refining MPE techniques and enhancing patient outcomes.

## CONFLICT OF INTEREST STATEMENT

The authors declare no conflicts of interest.
